# Workplace Inhalant Abuse in Adult Female: Brief Report

**DOI:** 10.1155/2011/905380

**Published:** 2011-09-28

**Authors:** Rohit Verma, Yatan Pal Singh Balhara, Smita N. Deshpande

**Affiliations:** Department of Psychiatry and Drug Deaddiction, Post Graduate Institute of Medical Education and Research, Dr Ram Manohar Lohia Hospital, New Delhi 110060, India

## Abstract

Inhalant abuse is the purposeful inhalation of intoxicating gases and vapors for the purpose of achieving an altered mental state. With its propensity for being yet an under-recognized form of substance use, being gateway to hard substances, cross-cultural penetration crossing socioeconomic boundaries, and causing significant morbidity and mortality in early ages, the prevention of inhalant misuse is a highly pertinent issue. This clinical report identifies a newer perspective in the emergence of inhalant abuse initiation. We report a case of an adult female with late onset of inhalant dependence developing at workplace and recommend for greater awareness, prevention, and management of this expanding substance abuse problem.

## 1. Introduction

Inhalants are volatile substances that produce chemical vapors that can be inhaled to induce a psychoactive, or mind-altering, effect. They encompass a broad range of chemicals that may have different pharmacological effects and are found in hundreds of different products, for example, include fuels, solvents, propellants, glues, adhesives, and paint thinners.

Inhaled substances are widely available, convenient, inexpensive, easily concealed, and legal for specific intended uses but are intentionally misused by abusers. Many of these qualities are important factors that promote use in a young age group. The initiation of inhalant abuse onset at adulthood is unreported as per our knowledge. We report a rare case of onset of inhalant dependence in an adult female at workplace.

## 2. Case History

A 43-year-old married female of middle socioeconomic status Hindu nuclear family with well-adjusted premorbid personality and no past/or family history of any psychiatric illness/or substance abuse, working as an assistant secretary in a government office since 15 years presented with 3 years history of inhaling typewriter fluid. The first time she inhaled it by accident while typing and liked its relaxing effect. Thereafter, occasionally she would inhale it mostly by mistake during work, but never took an active effort to persuade it. Three years back, her mother-in-law moved to their home and patient reported having occasional heated arguments with her. After one such event, patient went to work and found that the typewriter fluid provided relaxation to her mind, and her tension was reduced. Thereafter, whenever she would have an altercation with the family members, she would break away and inhale the fluid, which she started keeping at home. Over next 4-5 months, she started using it on a daily basis, even without having any immediate stressful situation. She would use 3-4 bottles every day, bringing them from workplace, where she would find no difficulty in obtaining it. Gradually she started reporting craving for it and would become irritable complaining of body ache, restlessness, reduced concentration, and disturbed sleep when not able to obtain the fluid. The family members initially felt happy that she was not retaliating to their arguments but later started becoming annoyed with her as she would not pay much attention to household work and would be frequently found lying alone in her room. Even after the mother-in-law went back, patient kept on continuing using the fluid. Gradually over the past 1 year, the frequency of altercations with husband and coworkers increased over minor matters. One week back when the patient disclosed her fluid inhalation to the husband, he brought her to the psychiatrist. A diagnosis of inhalant abuse was made, and patient was investigated with routine haematological and biochemical studies, liver/kidney function tests, B_12_ level, urine examination, radiographs of the chest, ECG, and brain CT imaging, which were normal. Mini international neuropsychiatric interview was insignificant for any other psychiatric disorder [1].

After psychoeducating regarding substance use and taking support from the husband as cotherapist, she was initiated on weekly counseling and motivational enhancement therapy. Currently, after 8 weeks of regular sessions, the patient is abstinent from the fluid and reports euthymic mood with integration in her usual routine activities.

## 3. Discussion

Inhalant abuse occurs throughout the world, in industrialized nations as well as developing countries ravaging the society in innumerable ways [2]. Usually inhalant use onset is during childhood or adolescence with declining pattern but can continue into adulthood [2]. Use by adults may predominate under particular circumstances, such as when certain occupations make abusable solvents, propellants, or anesthetics readily available. Data from the National Comorbidity Survey reveal that the aggregate prevalence of inhalant and other controlled substance abuse is as high as 7.5% in adults [3]. There is scarce data on inhalant use amongst adolescents and young adults from India [4]. This case highlights the importance of workplace, precipitating inhalant abuse onset in adult in lieu with stressful life circumstances.

A sizable chunk of employees in the organized sector is substance users, while others (nonsubstance users) remain vulnerable to substance use [5]. Substance use influences work adversely in the form of inconsistent work quality, poor concentration, lowered productivity, increased absenteeism, errors in judgment, interpersonal difficulties, frequent illnesses, devastating accidents, and financial losses [6]. Importantly, these adverse consequences of substance use are not only limited to “dependent” users but are seen in occasional users as well [6].

Thus, “workplace” appears to be an appropriate setting for interventions to prevent inhalant abuse amongst other substance use among those at risk. As the world is moving towards increased industrialization, there is an urgent need to replicate and scale-up workplace-based interventions at a wider level including, but not limited to, substance use. We suggest that the awareness for harmful effects of inhalant is required to be integrated within industrial health guidelines. Local manpower development for substance abuse cessation needs to be built to ensure continuation of assistance to the employees, and to encourage similar activities in nearby workplaces.

## 4. Conclusion

With mounting stress in society, the availability of a substance like inhalant needs to be addressed as certain characteristics of inhalants delineate it from other substances of abuse at workplace, important ones being easy procurement at workplace, unawareness about ill effects, no significant social awareness and restriction, no policy for inhalant abuse, and having similar pleasure effects as other substances of abuse.

##  Key Messages

1. Need to provide knowledge for harmful potential of inhalant use.
2. Concern to be raised about poor coping abilities with stressful life circumstances.
3. Need to targeting workplaces for avoiding potential inhalant abuse.
4. Focus on adult women as an emerging potential abuser.
